# Analysis of Common Beta-Thalassemia (β-Thalassemia) Mutations in East Java, Indonesia

**DOI:** 10.3389/fped.2022.925599

**Published:** 2022-07-15

**Authors:** Yetti Hernaningsih, Yuli Syafitri, Yulia Nadar Indrasari, Prafa Alif Rahmawan, Mia Ratwita Andarsini, Indra Lesmana, Emmanuel Jairaj Moses, Nur Arzuar Abdul Rahim, Narazah Mohd Yusoff

**Affiliations:** ^1^Department of Clinical Pathology, Faculty of Medicine Universitas Airlangga, Dr. Soetomo Academic General Hospital, Surabaya, Indonesia; ^2^Study Program of Clinical Pathology, Faculty of Medicine Universitas Airlangga, Surabaya, Indonesia; ^3^Department of Pediatrics, Faculty of Medicine Universitas Airlangga, Dr. Soetomo Academic General Hospital, Surabaya, Indonesia; ^4^Laboratory of Genetics and Breeding, Faculty of Biology, Universitas Gadjah Mada, Yogyakarta, Indonesia; ^5^Advanced Medical and Dental Institute, Universiti Sains Malaysia (USM), Penang, Malaysia

**Keywords:** β-thalassemia, β-thalassemia mutations, East Java, Indonesia, anemia

## Abstract

**Background:**

The frequency of the beta-thalassemia (β-thalassemia) gene in Indonesia ranges from 3 to 10%. However, in the East Java province, there is still limited information on the prevalence of β-thalassemia mutations in clinically diagnosed beta-thalassemia patients of East Java. Therefore, this study aimed to characterize β-thalassemia mutations in selected patients in the East Java province of Indonesia.

**Methods:**

This is an analytical observational study. Diagnosis of β-thalassemia was based on clinical presentation, complete blood count (CBC), and hemoglobin (Hb) electrophoresis. Blood specimens taken from each patient in three ethylenediaminetetraacetic acid (EDTA) tubes were analyzed for CBC and Hb electrophoresis and processed for DNA extraction and subsequent polymerase chain reaction (PCR). Detection of mutations in Hemoglobin Subunit Beta (HBB) gene exons 1–3 of the β-thalassemia gene as the common mutation in Indonesia was done using PCR followed by Sanger sequencing.

**Results:**

In total, 33 (*n* = 33) participants were involved in this study with ages ranging from 5 to 17 years comprising 19 women and 14 men. Their ethnic origins were Javanese (*n* = 30) and Chinese (*n* = 3). CBC results showed that mean ± standard deviation (SD) for Hb, red blood cell (RBC), mean corpuscular volume (MCV), mean corpuscular hemoglobin (MCH), mean corpuscular hemoglobin concentration (MCHC), and red cell distribution width (RDW)-CV were 81.2 ± 7.0 g/L; 3.40 ± 0.39 × 10^9^/L; 71.05 ± 5.72 fL; 24.12 ± 2.45 pg; 33.91 ± 1.47 g/dl; 24.38 ± 6.02%, respectively. Hb electrophoresis revealed that 5 out of 33 participants had beta-thalassemia and 28 out of 33 participants had hemoglobinopathy (Hb) E/beta-thalassemia. Results of Sanger sequencing showed the following genotype variations in the samples: 12 (36.4%) with β^*CD*26^*/*β^*IVS*−*I*−5^; 6 (18.2%) with β^*CD*26^*/*β^*CD*35^; 3 (9.1%) with β^*CD*26^*/*β^*IVS*−*I*−2^; 2 (6.1%) with β^*CD*27/28^*/*β^*CD*40^; 2 (6.1%) with β^*IVS*−*I*−1^*/*β^*CAP*+1^; and β^*CD*26^*/*β^*IVS*−*I*−1^; β^*IVS*−*I*−5^*/*β^*CAP*+1^; β^*IVS*−*I*−5^*/*β^*CD*35^; β^*CD*26^*/*β^*CD*37^; β^*CD*26^*/*β^*CD*15^; β^*CD*26^*/*β^*CD*40^; and β^*IVS*−*I*−5^*/*β^*CD*19^ in 1 (3%) sample, respectively, and 1 (3%) had no abnormality detected in sequencing even though electrophoresis showed abnormality in the migration pattern. The β^*CD*26^*/*β^*IVS*−*I*−5^ mutation was found in samples that were noted to have Hb E/beta-thalassemia on Hb electrophoresis.

**Conclusion:**

The underlying genetic variations are heterogeneous in thalassemia patients in East Java, where 12 variants were found. The most common variant was β^*CD*26^*/*β^*IVS*−*I*−5^, which all accounted for Hb E/beta-thalassemia on Hb electrophoresis. Furthermore, 28 out of 33 participants had hemoglobinopathy (Hb) E/beta-thalassemia.

## Introduction

Beta thalassemia (β-thalassemia) is a disorder in hemoglobin synthesis characterized by decreased or absent β-globin chain synthesis. There are two (2) groups of β-thalassemia based on the amount of β-globin chain synthesis, namely, β^0^ if the globin chain is not synthesized at all, and β+ thalassemia if the globin chain synthesis is reduced. β^0^ thalassemia is mainly caused by point mutations in the coding region or exon-intron junction of the β-globin gene that causes premature stop codons or causes abnormal β-globin mRNA (1).

More than 200 different mutations underlying β-thalassemia have been identified (2, 3). These mutations are divided into β-globin gene deletions and non-deletional mutations that affect the transcription, processing, or translation of the globin messenger. Point mutations include mutations at the Catabolite Gene Activator Protein (CAP) site, frameshift, initiation site, nonsense mutation, polyA addition site mutation, promoter, and splicing mutation (2).

Some of these mutations underlie the different clinical manifestations in the affected patients and are classified as transfusion-dependent (TD) and non-transfusion-dependent (NTD) thalassemia, based on the transfusion needs of a patient (3). Patients classified as NTD thalassemia (NTDT) may not require frequent blood transfusions for survival, whereas those with TD thalassemia (TDT) require life-long regular blood transfusions (4).

The epidemiology of thalassemia involves more than 150 countries in the world that includes the Mediterranean, certain parts of North and West Africa, the Middle East, the Indian subcontinent, Southern Far East, with Southeast Asia having the highest prevalence (1, 4). Indonesia is not spared, having a high frequency of those with thalassemia genes. This is evident from epidemiological studies in Indonesia, which found that the frequency of beta-thalassemia genes ranged from 3 to 10% (5). Every year about 300,000–500,000 newborns are accompanied by severe hemoglobin abnormalities and 50,000–100,000 children die from thalassemia; 80% of them reside in developing countries. Data obtained from all teaching hospitals only registered about 7,670 thalassemia major patients throughout Indonesia. This number is still much lower than the actual estimated number. This could be because the types of gene mutations that exist in Indonesia vary from very severe to mild, thus they do not require transfusion (asymptomatic), resulting in under-diagnosis (5).

The molecular basis of the thalassemia's has been studied in many of the world's population and studies pertaining to the molecular basis of thalassemia in Indonesia have been reported (6, 7), especially in East Java. However, in these earlier reports, there were a limited number of samples, and studies were conducted about 10 years ago. A preliminary study conducted on 17 patients with TDT revealed the presence of seven (7) genotypic variations of beta-thalassemia (6). Currently, in Indonesia, the number of patients reported having severe thalassemia is increasing annually, and these numbers have increased four-fold in the last 20 years (7).

One of the reasons for this increase in the number of patients with thalassemia is due to high population migration to and from East Java over the last 10 years resulting in an increase in inter-ethnic marriages (8, 9).

Dr. Hospital Soetomo is a referral and the largest hospital in eastern Indonesia, with a land area of 166,061 hectares and 1,444 beds. This hospital was established in 1950 and provides services, education, and research functions, obtaining the Joint Commission International (JCI) accreditation in 2018 (10). Regarding data on new patients with TDT, there were 51 patients in 2014, 69 patients in 2015, 39 patients in 2016, 47 patients in 2017, 41 patients in 2018, and 57 patients in 2019. Routine pediatric outpatient visits were around 187 per month (unpublished data, Hematology-Oncology Division-Department of Pediatrics, Dr. Soetomo General Academic Hospital, 2022).

In East Java province, there is limited information on the prevalence of β-thalassemia mutations in the affected population. Therefore, this study aimed to characterize β-thalassemia mutations in clinically diagnosed patients with beta-thalassemia TDT in the East Java province of Indonesia.

## Materials and Methods

### Patients and Study Design

The design of this study was analytical and observational. Participating patients were recruited from the One Day Care Unit of the Department of Pediatrics, Dr. Soetomo General Hospital, Surabaya, Indonesia. Previously diagnosed beta-thalassemia patients (<18 years old) who have undergone treatment that included blood transfusions at this institution were recruited. This study was carried out between July 2021 and January 2022.

Ethical clearance was obtained from the Ethics Committee of RSUD Dr. Soetomo No.: 0224/KEPK/VII/2021, and all of the patient's parents have agreed to give consent form.

### Laboratory Tests

Blood samples were taken from all patients on their follow-up prior to their blood transfusion. A total of 6 ml of blood was divided into two ethylenediaminetetraacetic acid (EDTA) tubes. The first tube was analyzed for complete blood count (CBC) using a Sysmex XN 1000 Analyzer (Sysmex Corporation, Kobe, Japan) and run for mini capillary hemoglobin electrophoresis (Sebia 9 Hydragel K20 Hemoglobin; Capillarys® Sebia, Lisses, France), while the other tube for DNA extraction using the QIAamp® DNA Blood Mini Kit lot no. kit. 166051764 (Qiagen GmbH, Hilden, Germany) according to the manufacturer's instructions. DNA samples were stored at −80°C, before subsequent analysis, i.e., performed polymerase chain reaction (PCR) and followed by the Sanger sequencing on these DNA samples. Ferritin, liver function tests, and renal function tests data were obtained from medical records based on the latest data. Molecular analysis was carried out at the Universitas Gajah Mada (UGM) Integrated Research and Testing Laboratory. Analytical statistics included the calculation of frequency distribution, mean, and standard deviation (SD).

### Genotype Determination

Extracted DNA samples were subjected to Sanger sequencing. In this PCR reaction, the mixture contained 12.5 μl (Bioline My Taq HS Red Mix), 50–150 ng of genomic DNA, and two pairs of primers (Table 1) at concentrations of 0.4 μM each. The PCR reactions were performed using Bio-Rad T100 (Bio-Rad Laboratories, Inc., USA). The PCR cycle reactions were as follows: an initial 2 min of denaturation at 95°C; 15 s of denaturation (35 cycles) at 95°C; 15 s of annealing at 54°C; 15 s of extension at 72°C; and 2 min of extension at 72°C. Subsequently, 5 μl of the amplified product was aliquoted prior to visualization using gel electrophoresis. The gel preparation was as follows: 1% agarose gel in 1 × Tris-Borate-EDTA buffer. Electrophoresis was conducted for 25 min at 100 V and then viewed under a UV transilluminator prior to documentation (11).

**Table 1:** Sequence and size of the primers used for DNA amplification (17).

**Primers**	**Sequence**	**Size (bp)**
Primer 1	5′ CCA AGG ACA GGT ACG GCT GTC ATC 3′	704 bp
Primer 5	5′ CCT TCC TAT GAC ATG AAC TTA ACA TT 3′	
Primer 6	5′ CTT TCC CTA ATC TCT TTC TTT CAG G 3′	470 bp
Primer 9	5′ GGA ACA AAG GAA CCT TTA ATA G 3′	

### DNA Sequence Analysis

The PCR products encompassing the β globin genes were amplified with double reaction PCR. The amplified fragments were subsequently sequenced by Sanger methods by DNA Sequencing Services (UGM Integrated Research and Testing Laboratory). The sequences were analyzed and aligned using the Benchling web service (https://www.benchling.com/) with the reference sequence from NCBI Reference Seq: NG_059281 to determine the genotype (Applied Biosystems, 3500 Genetic Analyzer, Hitachi Corp Tokyo, Japan).

## Results

There were thirty-three study participants (19 women and 14 men) wherein 29 were Javanese and 4 were Chinese involved in this study with ages ranging from 5 to 17 years. CBC results showed that mean ± SD for Hb, red blood count (RBC), mean corpuscular volume (MCV), mean corpuscular hemoglobin (MCH), mean corpuscular hemoglobin (MCHC), and red cell distribution width (RDW)-CV were 81.2 ± 7.0 g/L; 3.40 ± 0.39 × 10^9^/L; 71.05 ± 5.72 fL; 24.12 ± 2.45 pg; 33.91 ± 1.47 g/dl; and 22.91 ± 8.66%, respectively. The patients have hypochromic microcytic anemia with anisocytosis on the peripheral blood film, the results of transaminase were mildly increased, however, the mean result of renal function was still within the normal range (Table 2).

**Table 2:** Results of hematology, liver function test (LFT), ferritin, and renal function test (RFT) of all 33 patients.

**Parameter**	**Value**
Hematology laboratory, mean ± SD (min—max)	
Hemoglobin (g/L)	81.2 ± 7.0 (68.0–96.0)
RBC (x10^9^/L)	3.40 ± 0.39 (2.62–4.30)
MCV (fL)	71.05 ± 5.72 (60.00–86.30)
MCH (pg)	24.12 ± 2.45 (19.50–29.00)
MCHC (g/dL)	33.91 ± 1.47 (31.80–37.80)
RDW-CV	24.38 ± 6.02 (14.60–37.60)
WBC ( × 10^3^/L)	7.01 ± 1.98 (4.11–11.97)
PLT ( ×10^3^/L)	294.82 ± 138.65 (84.00–765.00)
Ferritin (ng/ml)	2564.39 ± 1616.40 (218.70–7109.30)
Serum glutamic oxaloacetic transaminase (SGOT) (U/L)	44.61 ± 24.26 (20.00–128.00)
Serum glutamic pyruvic transaminase (SGPT) (U/L)	39.52 ± 33.06 (12.00–149.00)
Blood urea nitrogen (BUN) (mmol/L)	3.26 ± 1.08 (0.71–5.71)
Serum creatinine (μmol/L)	33.59 ± 9.72 (8.84–53.04)

Hemoglobin electrophoresis revealed that five (5) out of 33 patients had β-thalassemia and 28 out of 33 participants had hemoglobin variant (Hb) E/β-thalassemia. Results of Sanger sequencing showed the following genotype variations: 12 (36.4%) with β^*CD*26^*/*β^*IVS*−*I*−5^; 6 (18.2%) with β^*CD*26^*/*β^*CD*35^; 3 (9.1%) with β^*CD*26^*/*β^*IVS*−*I*−2^; 2 (6.1%) with β^*CD*27/28^*/*β^*CD*40^; 2 (6.1%) with β^*IVS*−*I*−1^*/*β^*CAP*+1^; and β^*CD*26^*/*β^*IVS*−*I*−1^*;* β^*IVS*−*I*−5^*/*β^*CAP*+1^*;* β^*IVS*−*I*−5^*/*β^*CD*35^*;* β^*CD*26^*/*β^*CD*37^*;* β^*CD*26^*/*β^*CD*15^*;* β^*CD*26^*/*β^*CD*40^*;* and β^*IVS*−*I*−5^*/*β^*CD*19^ in each sample (3%), respectively, while one (1) sample (3%) had no mutation detected even though the Hb electrophoresis results showed abnormality in the migration pattern.

There were 12 mutations detected, with β^*CD*26^*/*β^*IVS*−*I*−5^ being the most common identified, followed by β^*CD*26^*/*β^*CD*35^ and β^*CD*26^*/*β^*IVS*−*I*−2^, respectively (Table 3). All samples, which were identified to have the β^*CD*26^*/*β^*IVS*−*I*−5^ mutation, were noted to have Hb E/β-thalassemia on Hb electrophoresis. These samples were from 11 Javanese and 1 Chinese patient.

**Table 3:** The frequency of β-thalassemia genotype variations.

**No**	**Genotype**	**Number of patients**	**Frequency (%)**
1	*β^*CD*26^/β^*IVS*−*I*−5^*	12	36.4
2	*β^*CD*26^/β^*CD*35^*	6	18.2
3	*β^*CD*26^/β ^*IVS*−*I*−2^*	3	9.1
4	*β^*CD*26^/β ^*IVS*−*I*−1^*	1	3
5	*β ^*IVS*−*I*−5^/ β ^*CAP*+1^*	1	3
6	*β ^*IVS*−*I*−5^/ β ^*CD*35^*	1	3
7	*β^*CD*26^/β^*CD*37^*	1	3
8	*β^*CD*26^/β^*CD*15^*	1	3
9	*β^*CD*27/28^/β^*CD*40^*	2	6.1
10	*β^*CD*26^/β^*CD*40^*	1	3
11	*β ^*IVS*−*I*−5^/ β ^*CD*19^*	1	3
12	*β ^*IVS*−*I*−1^/ β ^*CAP*+1^*	2	6.1

Patient nos. 17 and 18 were siblings and they had the same type of mutation but with different Hb electrophoresis results. Patient no. 18 who had HbE/β-thalassemia had lower Hb, MCV, MCH, and mean corpuscular hemoglobin concentration (MCHC) and higher RDW-CV as compared to patient no. 17 with β-thalassemia only, with results as follows: 81.0 vs. 94.0 g/L, 69.10 vs. 70.50 fL, 23.10 vs. 24.50 pg, 33.50 vs. 34.80 g/dl, 23.70 vs. 21.90, respectively (Table 4).

**Table 4:** Biodata, ethnicity groups, and results of hemoglobin electrophoresis, genotyping of β-thalassemia gene, and GenBank accession number in all 33 patients.

**ID**	**Sex**	**Age (year)**	**Ethnic**	**Hemoglobin electrophoresis**	**Genotype**	**GenBank Accession Number https://www.ncbi.nlm.nih.gov/genbank/**,
1	M	12	Javanese	Hb E/Beta Thalassemia	*β ^*IVS*−*I*−5^/β^*CAP*+1^*	ON584430
2	F	7	Javanese	Beta Thalassemia	*β ^*IVS*−*I*−5^/β^*CD*35^*	ON584431
3	M	17	Javanese	Hb E/Beta Thalassemia	*β^*CD*26^/β^*IVS*−*I*−5^*	ON584432
4	M	15	Javanese	Hb E/Beta Thalassemia	*β^*CD*26^/β^*IVS*−*I*−2^*	ON584433
5	F	17	Javanese	Hb E/Beta Thalassemia	*β^*CD*26^/β^*CD*35^*	ON584434
6	M	10	Javanese	Hb E/Beta Thalassemia	*β^*CD*26^/β^*CD*35^*	ON584435
7	F	11	Javanese	Hb E/Beta Thalassemia	*β^*CD*26^/β^*IVS*−*I*−5^*	ON584436
8	F	14	Javanese	Hb E/Beta Thalassemia	*β^*CD*26^/β^*IVS*−*I*−5^*	ON584437
9	F	9	Javanese	Hb E/Beta Thalassemia	*β^*CD*26^/β^*IVS*−*I*−5^*	ON584438
10	F	14	Javanese	Hb E/Beta Thalassemia	*β^*CD*26^/β^*IVS*−*I*−5^*	ON584439
11	M	13	Javanese	Hb E/Beta Thalassemia	*β^*CD*26^/β^*CD*35^*	ON584440
12	F	9	Javanese	Hb E/Beta Thalassemia	*β^*CD*26^/β^*CD*37^*	ON584441
13	F	15	Javanese	Hb E/Beta Thalassemia	*β^*CD*26^/β^*CD*15^*	ON584442
14	M	10	Javanese	Hb E/Beta Thalassemia	*β^*CD*26^/β^*IVS*−*I*−5^*	ON584443
15	F	14	Javanese	Hb E/Beta Thalassemia	*β^*CD*26^/β^*IVS*−*I*−5^*	ON584444
16	M	12	Javanese	Hb E/Beta Thalassemia	*β^*CD*26^/β^*IVS*−*I*−2^*	ON584445
17	F	10	Chinese	Beta Thalassemia	*β^*CD*27/28^/β^*CD*40^*	ON584446
18	M	12	Chinese	Hb E/ Beta Thalassemia	*β^*CD*27/28^/β^*CD*40^*	ON584447
19	F	11	Javanese	Hb E/Beta Thalassemia	*β^*CD*26^/β^*CD*35^*	ON584448
20	F	5	Javanese	Beta Thalassemia	*β ^*IVS*−*I*−1^/β^*CAP*+1^*	ON584449
21	M	14	Javanese	Beta Thalassemia	*β ^*IVS*−*I*−1^/β^*CAP*+1^*	ON584450
22	M	11	Javanese	Hb E/Beta Thalassemia	*β^*CD*26^/β^*IVS*−*I*−2^*	ON584451
23	M	8	Javanese	Hb E/Beta Thalassemia	*β^*CD*26^/β^*IVS*−*I*−5^*	ON584452
24	F	11	Javanese	Hb E/Beta Thalassemia	*β^*CD*26^/β^*IVS*−*I*−5^*	ON584453
25	F	12	Javanese	Hb E/Beta Thalassemia	*β^*CD*26^/β^*IVS*−*I*−1^*	ON584454
26	F	14	Javanese	Hb E/Beta Thalassemia	*β^*CD*26^/β^*CD*40^*	ON584455
27	M	9	Javanese	Beta Thalassemia	*β ^*IVS*−*I*−5^/β^*CD*19^*	ON584456
28	M	15	Chinese	Hb E/Beta Thalassemia	*β^*CD*26^/β^*IVS*−*I*−5^*	ON584457
29	F	8	Javanese	Hb E/Beta Thalassemia	*β^*CD*26^/β^*CD*35^*	ON584458
30	F	6	Javanese	Hb E/Beta Thalassemia	Not found mutation	ON584459
31	M	8	Javanese	Hb E/Beta Thalassemia	*β^*CD*26^/β^*IVS*−*I*−5^*	ON584460
32	F	14	Javanese	Hb E/Beta Thalassemia	*β^*CD*26^/β^*IVS*−*I*−5^*	ON584461
33	F	7	Javanese	Hb E/Beta Thalassemia	*β^*CD*26^/β^*IVS*−*I*−5^*	ON584462

*M, male; F, female. GenBank accession number was obtained after depositing the nucleotide sequences into GenBank. The datasets presented in this study can be found in online repositories. The names of the repository/repositories and accession numbers can be found below: https://www.ncbi.nlm.nih.gov/genbank/, ON584430–ON584462*.

A total of 66 alleles were analyzed, 12 types of mutations from 64 alleles were found, while 2 alleles from one sample were not detected. Twelve types of mutations include mutations with frequency as follows: *CD26 (G*>*A)* 25 (40.63%), *IVS-1-5 (G*>*C)* 15 (23.44%), *CD35 (delC)* 7 (11%), *IVS-1-2 (T*>*C); CAP*+*1 (A*>*C); CD40 (-G)* 3 (4.7%), respectively, *IVS-1-1 (G*>*T/A)* 3 (4.7%); *CD27/28* 2 (3.1%), and *CD37; CD15;* and *CD19* 1 (1.6%), respectively.

## Discussion

We reported the genotype variation in 33 patients with TDT patients at Dr. Soetomo General Hospital, Surabaya, Indonesia. Hemoglobin Subunit Beta (HBB) genes were determined on exons 1–3 based on the most frequent abnormality in the Java population and had proven that 32 out of the 33 patients had their genotype successfully identified. The results showed that 12 genotype variations were found with the highest frequency found in the *CD26/IVS-I-5* genotype (36.45%). This mutation was found in samples that have Hb E/beta-thalassemia on Hb electrophoresis and comprised 11 Javanese participants and one Chinese participant.

A previous study in East Java (6) showed a similar finding, which showed that 16 out of the 17 patients with TDT were compound heterozygotes for Hb E/beta-thalassemia (94.1%). The frequency calculated on the 34 independent alleles revealed Hb E 47.0%, *IVS-I-5* 20.6%; *CD35* 17.6%; *CD15 (–T)* 5.9%; *CD41/42* 2.9%; *IVS-II-654* 2.9%; and *PolyA* 2.9%.

The first report on beta-thalassemia genotype in Indonesia was by Lie-Injo (7). Out of 36 patients, 23 had β-thalassemia major and 13 had Hb E/beta-thalassemia with *CD26* mutation (7) of which the results are comparable to our study.

In a previous study by Rujito et al. (12) regarding the genotype distribution of beta-thalassemia in the Javanese population of the south region of Central Java, in 209 patients, genotypes were as follows: *CD26 /IVSI-5* (40.67%), followed by *IVS-I-5/IVS-I-5* (14.83%). Of the 418 known alleles that were examined, *IVS-I-5* was the most prevalent at 43.5% (12), which is comparable to our study.

The results of our study are also comparable to a recent study reported in Yogyakarta Special Region, conducted on 28 patients which showed six (6) β-globin gene mutations with *IVS-1-5 (G*>*C)* being the most dominant (71.4%) (13).

Another study in Bandung showed eight (8) types of mutations in 291 samples, with the highest prevalence of four (4) mutations, which were homozygous *IVS1nt5* 47.4%, heterozygote *IVS1nt5/…* (no paired mutation) 14.4%, heterozygous *IVS1nt5*/HbE 9.9%, and heterozygous *IVS1nt5/IVS1nt1* 5.4% (14). These results were slightly different from our study findings. This could be due to the different geographical locations of Java, which was West Java whereas our study was conducted in East Java.

A study conducted on 180 adolescent schoolgirls from East Java and West Java (15) found five (5) types of ß-globin gene polymorphism that were *CD2, CD26/*HbE*, IVS1nt5, IVS2nt16*, and *IVS2nt74*, which were quite similar in findings to our study; nevertheless *CD2, IVS2nt16*, and *IVS2nt74* were not found in our cohort.

In another recent study on 31 beta-thalassemia patients from East Kalimantan, which predominantly consisted of Javanese (64.5%), the results revealed seven (7 types) mutant alleles, which were *CD26/*HbE at 48.4%, *IVS-1-5* at 14.5%, *IVS-1-2* at 12.9%, *CD35* at 8.1%, and *IVS-1-1* at 6.5%. These were partially comparable to our results especially *CD26/*HbE*, IVS-1-2*, and *IVS-1-5* (16).

In comparison to other studies, our results revealed more mutations. It may reflect the variation of beta-globin mutation in the East Java population, since Dr. Soetomo Hospital is the biggest hospital in East Java and has become a referral hospital in East Java and eastern Indonesia. The results are fairly similar because West Java, Central Java, East Java, and the Special Region of Yogyakarta are all on one island, Java. Although East Kalimantan is part of Borneo island, i.e., a different island, the research was conducted in an area where the population is immigrants from Java, thus the results are almost the same.

Interestingly, 2 patients in our study were siblings (Table 4), and the CBC results in the sibling with HbE/β-thalassemia were much reduced than the sibling who had only β-thalassemia. This may be due to the fact that those with compound heterozygotes β-thalassemia/HbE have more severe clinical manifestations than those having only β-thalassemia. While one of them had no HbE detected in the Hb electrophoresis, both genotypes are identical, i.e., *CD27/28/CD40*. The explanation for this is that it may be related to the number of inherited *CD27/28* alleles, where a small number of alleles will produce low HbE and cannot be detected on electrophoresis.

Noteworthily, there was no mutation detected in one of the patients *via* Sanger sequencing. Nevertheless, this patient was transfusion dependent thus indicating a severe form of thalassemia. This finding highlights the limitation of this study as it only targeted exons 1–3 of the *HBB* gene. It is also not feasible to sequence all 20 exons of this gene *via* Sanger sequencing. Therefore, this scenario creates a need for a more effective and high throughput technology in the form of Next-Generation Sequencing (NGS) technology, which would greatly aid in unraveling the entire spectrum of beta-thalassemia mutations in the Indonesian population. Furthermore, a larger cohort would give a more accurate picture of the underlying molecular spectrum of mutations in patients with TD and NTD beta-thalassemia in Indonesia.

## Conclusion

The underlying genetic variations are heterogeneous in patients with TDT of East Java where there were 12 variants found, the most common of which is β^*CD*26^*/*β^*IVS*−*I*−5^, found in all patients with Hb E/beta-thalassemia detected *via* Hb electrophoresis.

The results of this study provide data that may be useful for the prevention and control strategies of thalassemia in Indonesia.

## Data Availability Statement

The datasets presented in this study can be found in online repositories. The names of the repository/repositories and accession numbers can be found below: https://www.ncbi.nlm.nih.gov/genbank/: ON584430, ON584431, ON584432, ON584433, ON584434, ON584435, ON584436, ON584437, ON584438, ON584439, ON584440, ON584441, ON584442, ON584443, ON584444, ON584445, ON584446, ON584447, ON584448, ON584449, ON584450, ON584451, ON584452, ON584453, ON584454, ON584455, ON584456, ON584457, ON584458, ON584459, ON584460, ON584461, ON584462.

## Ethics Statement

The studies involving human participants were reviewed and approved by Ethics Committee of RSUD Dr. Soetomo with No.: 0224/KEPK/VII/2021. Written informed consent to participate in this study was provided by the participants' legal guardian/next of kin.

## Author Contributions

YH designed and wrote the manuscript. YS collected samples and processed until DNA extraction, then sent to UGM. YI assisted in handling Hb electrophoresis, complete blood count. PR wrote the proposal to obtain funding. MA arrange for recruitment of pediatric patients. NY proofread and assisted with corrections until final version of manuscript. All authors contributed to the article and approved the submitted version.

## Funding

The authors thank the Faculty of Medicine Universitas Airlangga for funding the research, granted by Decree of the Chancellor of Airlangga University Number 212/UN3/2021.

## Conflict of Interest

The authors declare that the research was conducted in the absence of any commercial or financial relationships that could be construed as a potential conflict of interest. The handling editor ZAL declared a past co-authorship with the author NY.

## Publisher's Note

All claims expressed in this article are solely those of the authors and do not necessarily represent those of their affiliated organizations, or those of the publisher, the editors and the reviewers. Any product that may be evaluated in this article, or claim that may be made by its manufacturer, is not guaranteed or endorsed by the publisher.
